# Biochar Addition in Membrane Bioreactor Enables Membrane Fouling Alleviation and Nitrogen Removal Improvement for Low C/N Municipal Wastewater Treatment

**DOI:** 10.3390/membranes13020194

**Published:** 2023-02-04

**Authors:** Kanming Wang, Qiaoqiao Ye, Yuxiang Shen, Yajing Wang, Qiankun Hong, Chenlong Zhang, Min Liu, Hongyu Wang

**Affiliations:** 1 College of Architecture and Environment, Sichuan University, Chengdu 610000, China; 2 College of Environment, Zhejiang University of Technology, Hangzhou 310014, China; 3 Ningbo Communications Planning Institute Co., Ltd., Ningbo 315100, China

**Keywords:** membrane fouling, biochar, microbial diversity, low C/N ratio, carbon source release

## Abstract

Membrane bioreactors (MBRs) are frequently used to treat municipal wastewater, but membrane fouling is still the main weakness of this technology. Additionally, the low carbon-nitrogen (C/N) ratio influent has been shown to not only increase the membrane fouling, but also introduce challenges to meet the effluent discharge standard for nitrogen removal. Herein, the authors addressed the challenges by adding cost-effective biochar. The results suggested that the biochar addition can enable membrane fouling alleviation and nitrogen removal improvement. The reduced membrane fouling can be ascribed to the biochar adsorption capacity, which facilitates to form bigger flocs with carbon skeleton in biochar as a core. As a result, the biochar addition significantly altered the mixed liquor suspension with soluble microbial product (SMP) concentration reduction of approximately 14%, lower SMP protein/polysaccharide ratio from 0.28 ± 0.02 to 0.22 ± 0.03, smaller SMP molecular weight and bigger sludge particle size from 67.68 ± 6.9 μm to 113.47 ± 4.8 μm. The nitrogen removal is also dramatically improved after biochar addition, which can be due to the initial carbon source release from biochar, and formation of aerobic–anaerobic microstructures. Microbial diversity analysis results suggested more accumulation of denitrification microbes including *norank_f__JG30-KF-CM45* and *Plasticicumulans*. Less relative abundance of *Aeromonas* after biochar addition suggested less extracellular polymer substance (EPS) secretion and lower membrane fouling rate.

## 1. Introduction

Membrane bioreactor (MBR) is becoming an appealing alternative technology to conventionally activated sludge processes, due to the advantages of excellent effluent quality, complete control of hydraulic retention time (HRT) and solids retention time (SRT), and small footprint requirement [1,2]. However, membrane fouling control sets back the wide application and development of MBRs, resulting in the increased operational and maintenance cost [3].

In recent years, extensive endeavors have been made to alleviate membrane fouling, such as membrane modification, operational conditions optimization and mixed liquor suspension alternation [4]. The addition of adsorbents, such as activated carbon [5] and coagulants or flocculants [6], has been widely studied and could effectively control the membrane fouling. Powdered activated carbon (PAC) and granular activated carbon (GAC) have been broadly applied to MBR fouling alleviation, which can be explained by the reduced soluble microbial product (SMP) concentration as the high adsorption capacity of activated carbon for dissolved organic matter, enhanced scouring of the membrane surface and enlarged sludge floc size [7]. Lei et al. [8] indicated that PAC addition in anaerobic MBR mitigated the membrane fouling due to the restriction of cake layer formation. Sohn et al. [9] reported that PAC addition not only reduces the SMP and extracellular polymer substance (EPS) concentration, but also enhances the hydrophobicity and flocculation ability. Aslam et al. [10] suggested that GAC can provide the high specific area for biofilm formation and mechanical cleaning on membrane surface, therefore alleviating the membrane fouling. In comparison with the activated carbon, biochar seems to be more environmentally friendly and cost-effective. This is mainly due to the low pyrolysis temperature required for biochar synthesis from waste biomass, such as agricultural waste and waste-activated sludge without activation [1]. As a result, the price of BC was less than 10% of the PAC investment (USD 1.65–9.99 per kilogram) [11,12]. Furthermore, biochar has several functional groups in addition to the carbon backbone, with a substantial specific surface area, demonstrating an excellent adsorption ability of organic matters such as SMP [12]. Yet, aforementioned studies only highlighted the impact of the physiochemical property of the biochar, and the understanding of membrane fouling alleviation mechanism from the aspect of microbial diversity is still unclear.

With the implementation of a more stringent discharge standard, increasing attentions have been paid to the nitrogen removal in MBR via nitrification and denitrification processes [13,14]. Denitrification process is known to be conducted by heterotrophic microbes, that heavily rely on organic carbon as an electron donor [15]. However, it is a common problem that most of the wastewater treatment plants lack an adequate carbon source in the influent and some even have a low carbon–nitrogen ratio (C/N) of 3.8 [16]. The low C/N influent will not only be detrimental to the nitrogen removal due to the limited denitrification process [17], but also has a negative impact on the membrane fouling control [18]. Biochar is also known to have a certain amount of dissolved organic matter, and a previous study has indicated that the released carbon source can be conducive to the denitrification process [19]. Zhang et al. [20] investigated the impact of bamboo charcoal on MBR treatment performance and membrane fouling, and reported that bamboo charcoal addition can significantly improve the nitrogen removal and mitigate the membrane fouling. However, there is still a lack of understanding of the effect of biochar addition to submerged MBR for low C/N municipal wastewater treatment, focusing on the membrane fouling alleviation and nitrogen removal improvement.

The purpose of this study is to: (i) evaluate the impact of biochar addition to MBR treatment performance, especially for nitrogen removal; (ii) investigate the change of a mixed-liquor suspension and membrane fouling rate after biochar addition; (iii) elucidate the mechanism of biochar addition to control membrane fouling and improve nitrogen removal from the perspective of a microbial community.

## 2. Materials and Methods

### 2.1. Experiment Rig Set-Up and Operating Conditions

**Figure 1:**
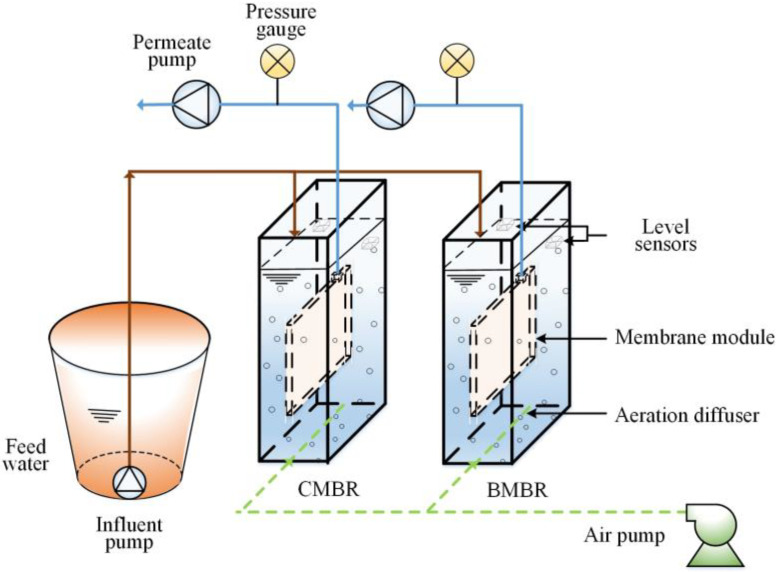
The schematic diagram of the experiment rig (CMBR: control MBR, BMBR: biochar MBR).

Two 6.5-L aerobic MBRs were set-up (Figure 1), with sludge inoculated from the aeration tank at a local sewage treatment plant (Hangzhou, China). The mixed liquor suspended solids (MLSS) concentration in the MBR was maintained at about 8500 mg/L. One gram per liter of biochar was added into one MBR (BMBR) and the other MBR was operated as control (CMBR). The biochar dosage was selected based on previous studies and from the economic perspectives [21]. These two MBR reactors were operated at the identical HRT for 6.2 h and SRT for 30 d. The reactor temperature was around 25.0 ± 1.5 °C during the trial. Synthetic sewage with a low C/N ratio of five was fed to the above two MBR reactors and the recipe was listed (Table S1 in Supplementary Materials).

A Dafu flat sheet (FS) polyvinylidene fluoride (PVDF) membrane was used (0.05 m^2^ surface area and 0.1 μm pore size, Jiangsu, China). Two membrane permeate pumps (BT100-2J, Longer Pump, Baoding, China) were operated for 9.0 min, followed by 1.0 min relaxation time. The membrane permeate flux was 21 L/(m^2^∙h). Air was introduced from the base of the reactor by a bubble strip (Boyu, Guangdong, China) to keep the dissolved oxygen (DO) in the mixed liquor suspension at 2.0–3.0 mg/L. The transmembrane pressures (TMP) were monitored and recorded by pressure gauges (Aosheng, Beijing, China) connecting to a data logger.

### 2.2. Biochar Characteristics

The coal biochar (average particle size of 0.154 mm) was purchased from a commercial company (Hongzhiyuan water purification, Henan, China). The morphology characteristics, specific area, and functional groups of the biochar were examined by scanning electron microscope (SEM) (Tecnai G2 F30 S-Twin, Philips-FE, Amsterdam, The Netherlands), chemisorption analyzer (ASAP 2460, Micromeritics, Atlanta, GA, USA) and Fourier transform infrared spectroscopy (FTIR) (Nicolet IS 10, Hi-Tech, Waltham, MA, USA) (Figures S1 and S2, Table S2 in Supplementary Materials). The pristine biochar and biochar in the MBR reactor after the trial were observed through microcopy (DN-10, Novel, Zhejiang, China).

### 2.3. Biochar Carbon Source Release Experiment

The carbon source release experiment of the biochar was conducted by adding 0.5 g biochar into an Erlenmeyer flask with 50 mL deionized water. The Erlenmeyer flask was shaken in a thermostatic oscillator (THZ-C, Suzhou, Jiangsu, China) at 150 rpm and 25 °C for 12 h, 24 h, 48 h, 72 h, 96 h, 120 h, 144 h, and 168 h. The mixture was sampled and centrifuged at 5000 rpm for 10 min (TG16-WS, Xiangyi, Hunan, China). The equal volume of deionized water was subsequently refilled back into the Erlenmeyer flask. The supernatants of the samples were filtered via 0.45 μm polyether sulfone (PES) filters (Jinteng, Tianjin, China) before analyses. Multiple parameters were scrutinized to analyze the supernatant, including chemical oxygen demand (COD), dissolved organic carbon (DOC), and volatile fatty acids (VFAs).

### 2.4. Membrane Fouling Analysis

The resistance-in-series model was utilized to assess the filtration resistances of a fouled membrane (Equation (1) and (2)) [22]. The total membrane filtration resistance (*Rt*, m^−1^) comprised an intrinsic membrane resistance (*Rm*, m^−1^), pore clogging resistance (*Rp*, m^−1^) and cake layer resistance (*Rc*, m^−1^):

(1) Rt=Rm+Rp+Rc

The Rt was determined based on Darcy’s law:

(2) $$Rt=\frac{TMP}{\mu J}$$

where *Rt* is the resistance (m^−1^), *TMP* is the transmembrane pressure (kPa), *J* is the permeate flux (L/(m^2^·h), and μ is permeate viscosity (Pa·s). Rm was determined by pure water permeability through the clean membrane. *Rt* was determined using the TMP at the end of membrane cycle. The fouled membrane was cleaned with deionized water (DI) to remove the fouling cake layer. The remaining resistance, after physical cleaning, was estimated as the sum of *Rp* and *Rm*. *Rp* can be calculated by subtracting *Rm*. After that, the membrane was cleaned with 500 mg/L sodium hypochlorite (NaClO) over night and the pure water permeability test was conducted to ensure permeability recovery before use [23].

### 2.5. Microbial Community Analyses

The mixed liquor suspensions from CMBR and BMBR were collected for microbial diversity analyses after the whole trial and stored at −20℃ until deoxyribonucleic acid (DNA) extraction, using the E.Z.N.A.^®^ Soil DNA Kit (Omega, Hartford, CT, USA). The primer of 338F-806R was used for DNA amplification. The 16S rDNA after amplification was sequenced and examined on Illumina Miseq PE300/NovaSeq PE250 platform by Majorbio (Shanghai, China).

### 2.6. Analytical Methods

COD, MLSS, ammonia (NH_4_^+^-N), nitrate (NO_3_^−^-N), nitrite (NO_2_^−^-N) and total nitrogen (TN) were measured following the standard methods [24]. The particle size distribution of the mixed-liquor suspension was measured by a LAP-W2000H particle size analyzer (Yishite, Xiamen, China). DOC was monitored by a TOC analyzer (Shimadzu-TOC-L-CPH/CPN, Tokyo, Japan). VFA was monitored through gas chromatography (Agilent 7890B, Santa Clara, CA, USA), with capillary column HP-INNOWax (30 m × 0.32 mm × 0.5 μm, Agilent, Santa Clara, CA, USA).

The sample preparation procedures of soluble microbial product (SMP) and extracellular polymeric substances (EPS) concentration can be referred to in our previous study [25]. All the supernatant samples were passed through 0.45 μm filters (Jinteng, Tianjin, China) prior to analyses. The SMP and EPS concentration was expressed as the sum of protein (PN) and polysaccharide (PS). The protein and polysaccharide concentrations were measured by a modified Lowry method [26] and phenol-sulfuric acid method, respectively [27]. The mixed-liquor suspensions were also characterized by three-dimensional excitation-emission matrix (3D-EEM) fluorescence spectroscopy (F-4700, Horiba Scientific, Kyoto, Japan). The SMP molecular weight (MW) fractionation was separated by an ultrafiltration cup (MSC300, Mosutech, Shanghai, China), with polyether sulfone (PES) membranes with different molecular weight cut-offs (MWCO) (100 kDa, 10 kDa and 1 kDa). The filtration was conducted at 0.2 MPa with pure nitrogen (>99%) stirred at 150 rpm by a magnetic stirrer.

All the experiments were conducted in triplicate and the statistical significance was tested by a Student’s *t*-test (SPSS 22.0), and the *p*-value < 0.05 was deemed statistically different.

## 3. Results and Discussion

### 3.1. Impact of Biochar Addition on Membrane Bioreactor Treatment Performance

Table 1 shows good COD and NH_4_^+^ removal in both CMBR and BMBR, with 96–98% and over 98%, respectively (Table S3 in Supplementary Materials). The low C/N ratio presented a challenge for both MBRs to achieve a high removal rate for NO_3_^−^-N or TN, because of the inadequate carbon source in the influent. It should be pointed out that even though approximately 62% of the TN was achieved, this can be ascribed to the limited oxygen diffusion into the floc providing oxygen-sufficient and oxygen-deficient zones, and facilitating nitrification and denitrification [28,29]. However, biochar addition to BMBR provided a significant improvement, which is sufficient to meet the First-class Level B requirement (20 mg/L TN) in China (GB18918-2002). The improved performance of BMBR can be attributed to the biochar addition with porous structure (Figure S1 in Supplementary Materials) and a high internal surface area of 645.667 m^2^/g [30] (Table S2 in Supplementary Materials), providing adsorption sites for microbes to form coexisting aerobic and anoxic microenvironment, and facilitate nitrogen-related metabolism [25].

Furthermore, biochar can initially provide extra carbon sources for denitrification microbes due to its release of carbon. In the carbon-source release experiment, after 192 h oscillation, the COD and DOC released from biochar are 7.0 ± 0.5 mg/g and 6.8 ± 0.7 mg/g, respectively (Table S4 in Supplementary Materials). This is comparable to an earlier study reporting that 10 mg/g DOC was released from rice husk biochar within 6 days [19]. In terms of VFA, the released acetic acid and propionic acid were identified with a concentration of 6.1 ± 0.6 mg/g and 5.0 ± 0.9 mg/g, respectively. It is worth noting that only biodegradable, organic compounds with weak binding diffused into water due to a concentration gradient, and the carbon source release due to the macromolecules hydrolysis into small soluble molecules by microorganisms should also be considered [31,32,33]. However, the carbon source from the biochar release is still limited, which may not fully surrogate the missing carbon source in the influent. Further studies about the biochar modification and carbon source release kinetics should be conducted in order to increase the carbon source content, control the release rate and fit the carbon source demand from the denitrification bacteria [34,35]. Nevertheless, this result suggested that the improved total nitrogen removal can also partially be ascribed to the initial carbon source release from the biochar.

### 3.2. Impact of Biochar Addition on Mixed Liquor Suspensions

**Figure 2:**
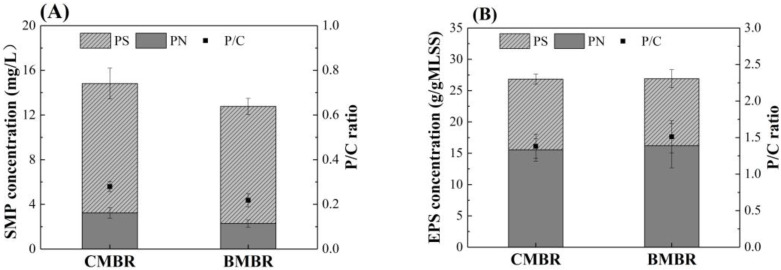
The compositions of (**A**) EPS and (**B**) SMP in the mixed-liquor suspension from conventional MBR (CMBR) and biochar MBR (BMBR).

SMP and EPS are main compounds that affect membrane fouling in the MBRs [36,37]. To further evaluate the adsorption capacity of biochar, SMP and EPS concentrations were measured in both MBRs. SMP concentration in BMBR (12.76 mg/L) was lower than CMBR (14.82 mg/L) (Figure 2A,B) (*p* < 0.05), which is primarily the consequence of reduced protein content. The average protein concentration in BMBR was 2.03 mg/L, which was about 30% less than that of CMBR. As a result, BMBR achieved lower SMP protein/carbohydrates (P/C) ratio of 0.22 ± 0.03 than that of 0.28 ± 0.02 in CMBR, indicating a lower fouling rate in BMBR, since proteins are more hydrophobic than carbohydrates and can easily bind to membranes [4]. In other words, the added biochar suppressed the SMP concentration in the bulk sludge in favor of lower membrane fouling propensity. This is consistent with Ye’s study, who also reported less SMP and lower membrane fouling with biochar addition, although with an anaerobic membrane bioreactor [38]. However, similar average EPS concentrations of 26.84 mg/L and 26.90 mg/L were observed in CMBR and BMBR, respectively. Results revealed that the biochar addition had no discernible impact on EPS concentration in the mixed-liquor suspension.

**Figure 3:**
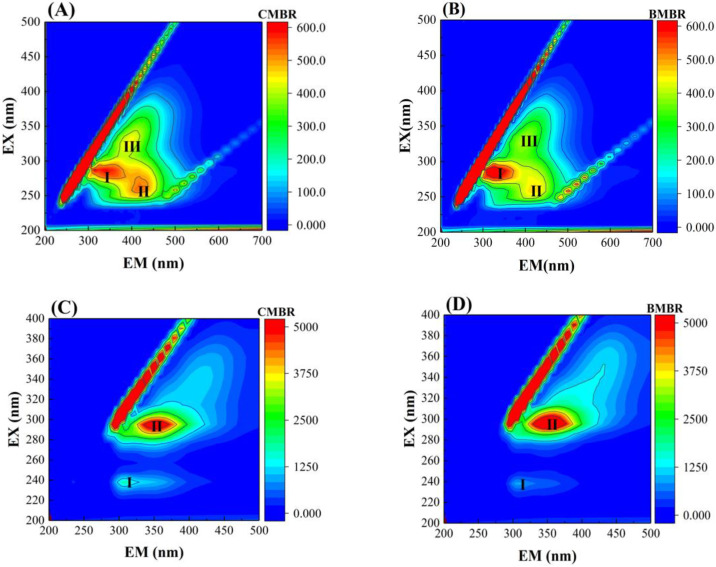
3D-EEM fluorescence spectra of SMP (**A**,**B**) and EPS (**C**,**D**) in the conventional MBR (CMBR) and biochar MBR (BMBR).

The 3D-EEM results further evaluated the impact of biochar addition on SMP and EPS composition in the bulk sludge (Figure 3). The 3D-EEM measurement of SMP had three peaks at excitation/emission wavelength (Ex/Em) of 275–300/300–380 nm (Peak I, tryptophan) and 250–275/400–450 nm, 280–350/375–425 nm (Peak II and Peak III, humic acid-like substances) [39,40]. BMBR had lower peak intensities compared to CMBR in Peak II and Peak III, indicating that the biochar addition could reduce the humic-like substances concentrations, and therefore mitigate membrane fouling since humic substances play a vital role in fouling formation [41]. In terms of EPS, peaks at Ex/Em of <250/<380 nm and 280–300/<380 nm were observed, suggesting aromatic proteins (Peak I) and tryptophan (Peak II), respectively (Figure 3A,B) [39,40]. There seems to be no obvious difference between the peak location and intensity for BMBR and CMBR, indicating that BMBR and CMBR had similar EPS compositions and concentrations.

**Figure 4:**
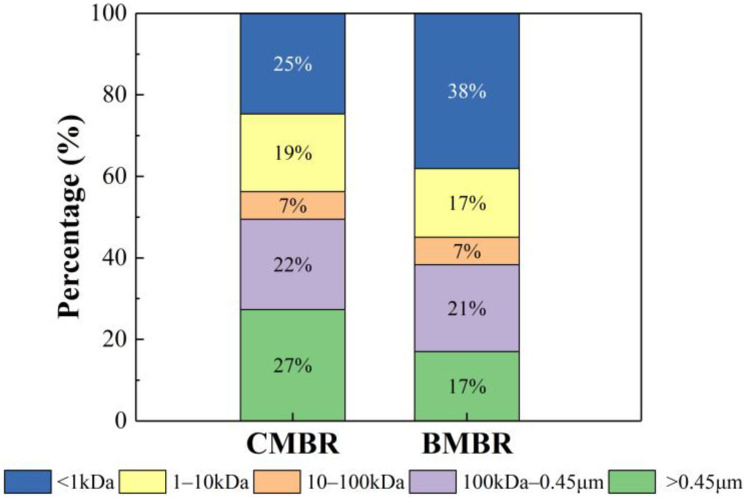
MW distributions of SMP in the mixed-liquor suspension from conventional MBR (CMBR) and biochar MBR (BMBR).

The MW distributions of SMP was further compared to evaluate the impact of biochar addition on fouling control (Figure 4). The proportions of macromolecules (MW > 0.45 μm and 100 kDa–0.45 μm) in BMBR (17% and 21%) was lower compared with those in CMBR (27% and 22%). In contrast, the small molecules (<1 kDa) occupied 38% of the total SMP in BMBR, which was higher than that of CMBR (25%). Previous studies suggested that macromolecules are more likely to be trapped on the membrane pore compared with small molecules, which can be used to explain the more serious membrane fouling in CMBR [36]. Interestingly, the biochar addition reduced the SMP macromolecules, which might be ascribed to the biodegradation due to attached microbes onto the biochar surface [42]. Zhang et al. [43] also discovered that biologically activated carbon formed by microbial attachment in the PAC-MBR system can make macromolecules organic matters into smaller size by microbial biodegradation. The reduction in SMP molecular weight could also be beneficial to membrane fouling control, as suggested by the Flory–Huggins’ theory [44], since small molecules have low cross-linking, free energy (chemical potential), and therefore lower fouling propensity.

The bulk sludge characterization, including particle size, zeta potential, settling property (SV30 and SVI), and capillary suction time (CST), were also conducted. Bigger median particle size (d50) of 113.47 ± 4.8 μm was observed in BMBR compared to that of 67.68 ± 6.9 μm in CMBR, indicating that biochar addition could increase the particle size of sludge (Table 2, Table S5 in Supplementary Materials). This may be due to the interaction between biochar and free bacteria and microbial flocs [45], forming the flocs with the carbon skeleton in biochar as the core (Figure S3 in Supplementary Materials). The reduced absolute value of zeta potential after biochar addition can also facilitate this bio-flocculation. The mixed-liquor suspension with a larger particle size also indicated lower membrane fouling propensity due to the increased shear-induced diffusion which making it more difficult to deposit onto the membrane [46]. As a result, larger sludge flocs have a better settling performance (Table 2). With the introduction of biochar, BMBR achieved a lower SV30 (78.9% ± 0.6%) compared to CMBR (87.5% ± 0.8%), and a lower SVI (90.5 ± 0.6 mL/g) compared to CMBR (128.1 ± 0.5 mL/g to 90.5 ± 0.6 mL/g). This would help to prevent the foaming issue in MBR operation and reduce the maintenance costs for sludge treatment [47,48].

### 3.3. Impact of Biochar Addition on Membrane Fouling Propensity and Fouling Mechanism

**Figure 5:**
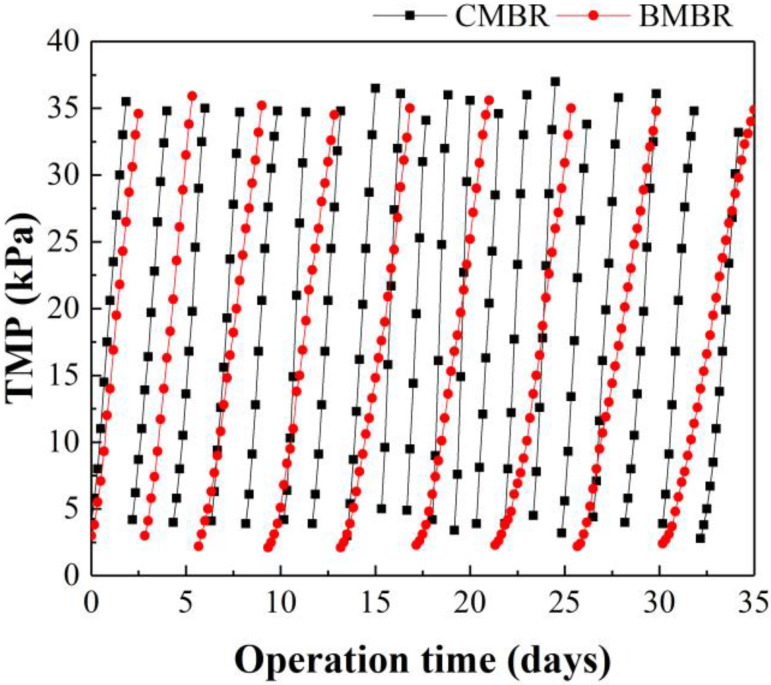
TMP profiles of the conventional MBR (CMBR) and biochar MBR(BMBR).

The TMP curves of the MBR with and without biochar addition were monitored (Figure 5). CMBR took 1.8 days (44 h) on average to reach the designated TMP limit of 35 kPa, whilst the BMBR extended this operation period to 3.9 days (94 h). This might be due to the adsorption capacity of biochar, suggested by its characterization results (Table S2 in Supplementary Materials), which decreased the SMP content in the mixed-liquor suspension. Similarly, Sima et al. [12] also reported that acid/alkali-modified biochar addition can successfully achieve membrane fouling mitigation. It must be pointed out that the relative short MBR operation period might be owing to the low C/N ratio, resulting in more SMP release since limited substances are available for the microbes [18,49]. Wang et al. [25] also reported that a low C/N influent could lead to more severe membrane fouling compared to a high C/N influent.

The distribution of membrane filtration resistances was also measured to characterize membrane foulant (Table 3). The cake layer resistance (Rc) of CMBR and BMBR is the key element of the filtration resistance. This is consistent with previous studies which also suggested that the membrane sludge cake layer representing the reversible fouling dominates the membrane filtration resistance [50,51]. The lower Rp/Rt from BMBR compared to CMBR was observed, indicating that BMBR exhibited lower irreversible fouling caused by pore clogging owing to the lower SMP concentration [51]. Similarly, previous researchers [25,51] also indicated that a higher SMP concentration has led to higher Rp/Rt proportions in the membrane fouling cake. The fouling cake was also monitored by FTIR and results indicated that fouling cake in CMBR and BMBR have similar functional groups (Figure S4 in Supplementary Materials). There is a large absorption region near the peak of 3400 cm^−1^, which is expressed as a hydroxyl (-OH) [52]. The peak near 2930 cm^−1^ represents a C–H bond, which is an olefin substance [53]. Three peaks at 1640 cm^−1^, 1550 cm^−1^ and 1400 cm^−1^ were also observed, which correspond to amide I, amide II and amide III, respectively. Additionally, a very obvious peak appears at 1068 cm^−1^, which is expressed as a polysaccharide substance [54]. In summary, the membrane fouling cake layer on both CMBR and BMBR has similar functional groups, revealing that proteins and polysaccharides are primary organic compounds of the membrane foulant.

### 3.4. Impact of Biochar Addition on Microbial Diversity

The microbial diversity was evaluated to assess the effect of biochar addition on microbial diversity with a low C/N influent. Alpha diversity is an important indicator to characterize the abundance and diversity of a microbial community. A Shannon diversity index is used to reflect the microbial diversity, and ACE and Chao indices are a typical microbial species richness estimator [55]. Table 4 showed higher indices of Shannon, ACE and Chao in BMBR compared to CMBR. There are also higher operational taxonomic units (OTUs) in BMBR with 890, compared with 776 in CMBR. The above microbial diversity change demonstrated that biochar addition could enrich the microbial population, diversity and richness.

**Figure 6:**
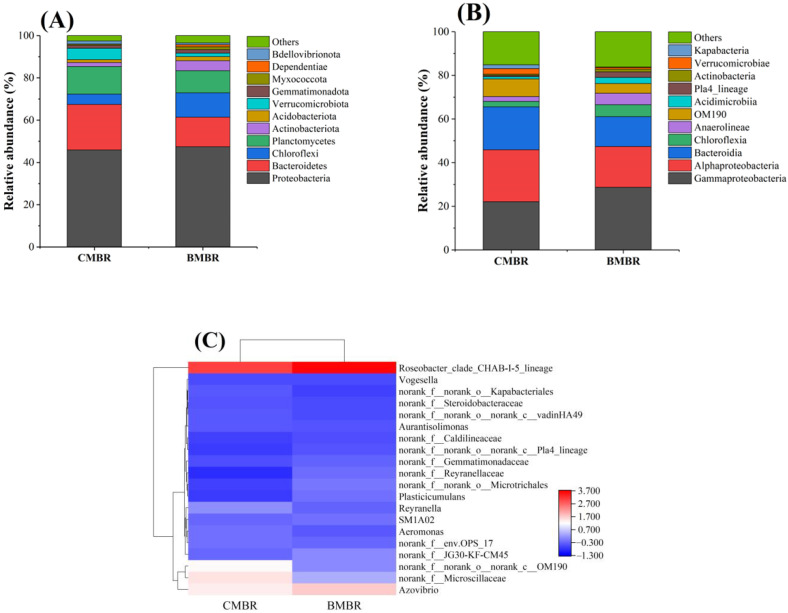
Microbial communities in the mixed-liquor suspension from conventional MBR (CMBR) and biochar MBR (BMBR) at (**A**) phylum (**B**) class and (**C**) genus level.

*Proteobacteria*, *Bacteroidetes*, *Planctomycetes* and *Chloroflexi* were most prominent at the phylum level in the bulk sludge, occupying over 83% of the total relative abundance (Figure 6A) [40]. *Proteobacteria* was the predominant phyla, occupying about 45.9% and 47.3% in CMBR and BMBR, respectively, which are widely reported with the function of nitrification as well as denitrification in typical sewage treatment facilities [56]. Significantly lower relative abundance of *Bacteroidetes* were observed in BMBR (14.0%) compared to that in CMBR (21.5%), suggesting less EPS protein secretion in the BMBR [57,58]. This was consistent with the lower SMP P/C ratio of the BMBR (Figure 2A), which revealed its lower membrane fouling tendency. The relative abundance of *Planctomycetes* in CMBR and BMBR were 13.1% and 10.5%, respectively. The lower abundance of *Planctomycetes* in BMBR can indicate lower fouling propensity since *Planctomycetes* is conducive for biofilm attachment onto membrane surfaces [58].

*Gammaproteobacteria*, *Alphaproteobacteria* and *Bacteroidia* were the most prevalent classes in both CMBR and BMBR (Figure 6B), accounting for 22.1%, 23.8% and 19.7% in CMBR, and 28.7%, 18.7% and 13.7% in BMBR, respectively. The enriched *Gammaproteobacteria* BMBR may facilitate nitrogen removal since it has excellent denitrification capability [59]. The relative abundance of *Anaerolineae* also enriched from 2.2% to 5.2%, which was regarded as the typical denitrification bacteria and can potentially explain the improved TN removal in BMBR [60]. Additionally, *Alphaproteobacteria*, which closely relate to membrane fouling [61], was more abundant in CMBR than BMBR, and indicated more severe membrane fouling in CMBR than BMBR.

The top 20 dominant genera in the CMBR and BMBR were displayed in a hierarchically clustered heatmap (Figure 6C). The microbial flora of *norank_f__JG30-KF-CM45* [62] and *Plasticucumulans* were more abundant in BMBR than in CMBR, which was identified as denitrification bacteria [63,64]. The relative abundance of *Plasticucumulans* differed drastically, from 2.3% in CMBR to 3.5% in BMBR, and may lead to better TN removal in BMBR. In addition, a lower relative abundance of Aeromonas was observed in BMBR compared with CMBR, indicating less EPS secretion and a lower membrane fouling rate [65].

## 4. Conclusions

The impact of the biochar addition in MBRs treating low C/N municipal wastewater was investigated. The following conclusions can be reached:

- Biochar addition can enable membrane fouling alleviation and nitrogen removal improvement.
- Biochar addition can effectively alleviate membrane fouling because of the change of mixed liquor, such as lower SMP content reduction by about 14%, lower SMP P/C ratio from 0.28 ± 0.02 to 0.22 ± 0.03, smaller SMP molecular weight and bigger particle size from 67.68 ± 6.9 μm to 113.47 ± 4.8 μm. This can be mainly ascribed to the biochar adsorption capacity and facilitate the formation of bigger flocs with the carbon skeleton in biochar as a core.
- The improved nitrogen removal after biochar addition can be due to the initial carbon source release from biochar and formation of aerobic–anaerobic microstructures.
- Microbial diversity analysis results suggested more accumulation of denitrification microbes, including *norank_f__JG30-KF-CM45* and *Plasticicumulans*. A less relative abundance of *Aeromonas* after biochar addition suggested less EPS secretion, thus reducing the rate of membrane fouling.
- Further studies about biochar modification and carbon source release kinetics should be conducted in order to increase the carbon source content, control the release rate and fit the carbon source demand from the denitrification bacteria. Additionally, the cost analyses of biochar addition on long-term performance should be carried out.

## Figures and Tables

**Table 1:** Effluent characteristics from the conventional MBR (CMBR) and biochar MBR (BMBR) feeding with synthetic municipal wastewater under low C/N ratio of five.

Parameter	CMBR		BMBR	
	Effluent Characteristics mg L^−1^	Removal %	Effluent Characteristics mg L^−1^	Removal %
COD	9.8 ± 0.9	96.4 ± 0.3	5.5 ± 0.9	97.9 ± 0.3
NH_4_^+^-N	0.8 ± 0.05	98.2 ± 0.1	0.7 ± 0.05	98.4 ± 0.1
NO_3_^−^-N	21.1 ± 1.5	-	16.7 ± 1.2 *	-
TN	21.9 ± 1.5	62.3 ± 0.5	17.4 ± 1.2 *	70.1 ± 0.6 *

* Statistical difference between CMBR and BMBR (*p* < 0.05).

**Table 2:** Characterization of mixed-liquor suspension from the conventional MBR (CMBR) and the biochar MBR (BMBR) at the end of the experimental trial.

Parameter	SVI (mL/g)	SV_30_ (%)	Particle Size (μm)	Zeta Potential (−mV)
CMBR	128.1 ± 6	98.0 ± 0.8	67.68 ± 6.9	20.6 ± 0.44
BMBR	90.5 ± 7	87.0 ± 0.8	113.47 ± 4.8	19.4 ± 0.30

**Table 3:** Membrane filtration resistance in conventional MBR (CMBR) and biochar MBR (BMBR).

	*Rt*	*Rm*	*Rp*	*Rc*
CMBR (×10^12^ m^−1^)	6.68	1.45	0.41	4.82
Percent of Rt	100%	21.74%	6.10%	72.16%
BMBR (×10^12^ m^−1^)	6.70	1.45	0.079	5.17
Percentage of Rt	100%	21.66%	1.18%	77.16%

**Table 4:** Richness and diversity index of the microbial community in conventional MBR (CMBR) and biochar MBR (BMBR).

Sample/Estimators	Shannon	Simpson	Ace	Chao	Pd	OTU
CMBR	4.426	0.043	863.633	863.065	84.134	776
BMBR	4.651	0.043	967.221	973.280	93.096	890

## Data Availability

The data presented in this study are available on request from the corresponding author.

## Supplementary Materials

The following supporting information can be downloaded at: https://www.mdpi.com/article/10.3390/membranes13020194/s1, Figure S1: Scanning electron microscopic (SEM) images of biochar; Figure S2: FTIR spectra and spectroscopic assignment of biochar; Figure S3: Microscope picture of (a) pristine BC and (b) the activated sludge with BC addition; Figure S4: FTIR spectra and spectroscopic assignment of the polluted membrane up to 35 kPa of conventional MBR (CMBR) and biochar MBR (BMBR); Table S1: Component of the synthetic wastewater; Table S2: BET surface area, micropore area, pore volume and pore diameter of biochar; Table S3: Treatment performance data and statistical results; Table S4: Amount of COD, DOC, SCFAs released from biochar; Table S5: Characterization of mixed-liquor suspension data.

## References

[1] Deng L., Guo W., Ngo H.H., Zhang X., Chen C., Chen Z., Cheng D., Ni S., Wang Q. (2022). Recent advances in attached growth membrane bioreactor systems for wastewater treatment. Sci. Total Environ..

[2] Xiao K., Liang S., Wang X., Chen C., Huang X. (2019). Current state and challenges of full-scale membrane bioreactor applications: A critical review. Bioresour. Technol..

[3] Bagheri M., Mirbagheri S.A. (2018). Critical review of fouling mitigation strategies in membrane bioreactors treating water and wastewater. Bioresour. Technol..

[4] Meng F., Chae S., Drews A., Kraume M., Shin H., Yang F. (2009). Recent advances in membrane bioreactors (MBRs): Membrane fouling and membrane material. Water Res..

[5] Aslam M., Charfi A., Lesage G., Heran M., Kim J. (2017). Membrane bioreactors for wastewater treatment: A review of mechanical cleaning by scouring agents to control membrane fouling. Chem. Eng. J..

[6] Wang H., Chen Z., Miao J., Li Y. (2016). A novel approach for mitigation of membrane fouling: Concomitant use of flocculant and magnetic powder. Bioresour. Technol..

[7] Remy M., Potier V., Temmink H., Rulkens W. (2010). Why low powdered activated carbon addition reduces membrane fouling in MBRs. Water Res..

[8] Lei Z., Yang S., Li X., Wen W., Huang X., Yang Y., Wang X., Li Y., Sano D., Chen R. (2019). Revisiting the effects of powdered activated carbon on membrane fouling mitigation in an anaerobic membrane bioreactor by evaluating long-term impacts on the surface layer. Water Res..

[9] Sohn W., Guo W., Ngo H.H., Deng L., Cheng D. (2021). Powdered activated carbon addition for fouling control in anaerobic membrane bioreactor. Bioresour.Technol. Rep..

[10] Aslam M., Yang P., Lee P.H., Kim J. (2018). Novel staged anaerobic fluidized bed ceramic membrane bioreactor: Energy reduction, fouling control and microbial characterization—ScienceDirect. J. Membr. Sci..

[11] Luo C., Lü F., Shao L., He P. (2015). Application of eco-compatible biochar in anaerobic digestion to relieve acid stress and promote the selective colonization of functional microbes. Water Res..

[12] Sima X.F., Wang Y.Y., Shen X.C., Jing X.R., Jiang H. (2017). Robust bio-charassisted alleviation of membrane fouling in MBRs by indirect mechanism. Sep. Purif. Technol..

[13] Chang M., Liang B., Zhang K., Wang Y., Jin D., Zhang Q., Hao L., Zhu T. (2022). Simultaneous shortcut nitrification and denitrification in a hybrid membrane aerated biofilms reactor (H-MBfR) for nitrogen removal from low COD/N wastewater. Water Res..

[14] Pynaert K., Smets B.F., Beheydt D., Verstraete W. (2004). Start-up of autotrophic nitrogen removal reactors via sequential biocatalyst addition. Environ. Sci. Technol..

[15] Dhamole P.B., Nair R.R., D’Souza S.F., Lele S.S. (2009). Simultaneous removal of carbon and nitrate in an airlift bioreactor. Bioresour. Technol..

[16] Wang Y., Geng J., Ren Z., Guo G., Wang C., Wang H. (2013). Effect of COD/N and COD/P ratios on the PHA transformation and dynamics of microbial community structure in a denitrifying phosphorus removal process: Nutrient ratios effect on PHA transformation in a A 2 N-SBR. J. Chem. Technol. Biotechnol..

[17] Han F., Ye W., Wei D., Xu W., Du B., Wei Q. (2018). Simultaneous nitrification-denitrification and membrane fouling alleviation in a submerged biofilm membrane bioreactor with coupling of sponge and biodegradable PBS carrier. Bioresour. Technol..

[18] Hao L., Liss S.N., Liao B.Q. (2016). Influence of COD:N ratio on sludge properties and their role in membrane fouling of a submerged membrane bioreactor. Water Res..

[19] Sima X.F., Li B.B., Jiang H. (2017). Influence of pyrolytic biochar on settleability and denitrification of activated sludge process. Chin. J. Chem. Phys..

[20] Zhang W., Liu X., Wang D., Jin Y. (2017). Effects of bamboo charcoal on fouling and microbial diversity in a flat-sheet ceramic membrane bioreactor. Bioresour. Technol..

[21] Jamal Khan S., Visvanathan C., Jegatheesan V. (2012). Effect of powdered activated carbon (PAC) and cationic polymer on biofouling mitigation in hybrid MBRs. Bioresour. Technol..

[22] Lee J., Ahn W., Lee C. (2001). Comparison of the filtration characteristics between attached and suspended growth microorganisms in submerged membrane bioreactor. Water Res..

[23] Wang K.M., Zhang L.J., Zhang H.L., Li J.L., Zhang Y.C., Liu B.C., Wang H.Y. (2021). Membrane fouling amelioration through pseudo dead-end filtration coupled with transmembrane pressure (TMP) set-point control in an anaerobic membrane bioreactor for municipal wastewater treatment. Environ. Sci.-Water Res. Technol..

[24] APHA (2005). Standard Methods for the Examination of Water and Wastewater.

[25] Wang K.M., Jiang S.F., Zhang Z.H., Ye Q.Q., Zhang Y.C., Zhou J.H., Hong Q.K., Yu J.M., Wang H.Y. (2020). Impact of static biocarriers on the microbial community, nitrogen removal and membrane fouling in submerged membrane bioreactor at different COD:N ratios. Bioresour. Technol..

[26] Lowry O., Rosenburgh N., Farr A. (1951). Protein measurements with the folin-phenol reagent. J. Biol. Chem..

[27] Dubois M., Gilles K.A., Hamilton J.K., Rebers P.A., Smith F. (1951). Colorimetric method for determination of sugars and related substances. Anal. Chem..

[28] Mao X., Myavagh P.H., Lotfikatouli S., Hsiao B.S., Walker H.W. (2020). Membrane bioreactors for nitrogen removal from wastewater: A review. J. Environ. Eng..

[29] Sarioglu M., Insel G., Artan N., Orhon D. (2009). Model evaluation of simultaneous nitrification and denitrification in a membrane bioreactor operated without an anoxic reactor. J. Membr. Sci..

[30] Foereid B. (2015). Biochar in nutrient recycling—The effect and its use in wastewater treatment. Open J. Soil. Sci..

[31] Fu X., Hou R., Yang P., Qian S., Feng Z., Chen Z., Wang F., Yuan R., Chen H., Zhou B. (2022). Application of external carbon source in heterotrophic denitrification of domestic sewage: A review. Sci. Total. Environ..

[32] He X., Zhang S., Jiang Y., Li M., Yuan J., Wang G. (2021). Influence mechanism of filling ratio on solid-phase denitrification with polycaprolactone as biofilm carrier. Bioresour. Technol..

[33] Yu L., Cheng L., Peng Z., Li T., Liu L., Liu X., Fan P. (2020). Carbon release mechanism of synthetic and agricultural solid carbon sources. Water Environ. J..

[34] Li C., Wang H., Yan G., Dong W., Chu Z., Wang H., Chang Y., Ling Y., Zhang Y. (2022). Initial carbon release characteristics, mechanisms and denitrification performance of a novel slow release carbon source. J. Environ. Sci..

[35] Weng Z., Ma H., Ma J., Kong Z., Shao Z., Yuan Y., Xu Y., Ni Q., Chai H. (2022). Corncob-pyrite bioretention system for enhanced dissolved nutrient treatment: Carbon source release and mixotrophic denitrification. Chemosphere.

[36] Deng L., Guo W., Ngo H.H., Zhang H., Wang J., Li J., Xia S., Wu Y. (2016). Biofouling and control approaches in membrane bioreactors. Bioresour. Technol..

[37] Meng F., Zhang S., Oh Y., Zhou Z., Shin H., Chae S. (2017). Fouling in membrane bioreactors: An updated review. Water Res..

[38] Ye L., Xia T., Chen H., Ling L., Xu X., Alvarez P.J.J., Zhu L. (2018). Effect of bamboo charcoal amendment on an AnMBR in the aspect of anaerobic habitat and membrane fouling. Environ. Sci. Water Res. Technol..

[39] Chen W., Westerhoff P., Leenheer J.A., Booksh K. (2003). Fluorescence excitation−emission matrix regional integration to quantify spectra for dissolved organic matter. Environ. Sci. Technol..

[40] He Q., Song Q., Zhang S., Zhang W., Wang H. (2018). Simultaneous nitrification, denitrification and phosphorus removal in an aerobic granular sequencing batch reactor with mixed carbon sources: Reactor performance, extracellular polymeric substances and microbial successions. Chem. Eng. J..

[41] Jiang W., Xia S., Liang J., Zhang Z., Hermanowicz S.W. (2013). Effect of quorum quenching on the reactor performance, biofouling and biomass characteristics in membrane bioreactors. Water Res..

[42] Hazrati H., Jahanbakhshi N., Rostamizadeh M. (2018). Fouling reduction in the membrane bioreactor using synthesized zeolite nano-adsorbents. J. Membr. Sci..

[43] Zhang S., Xiong J., Zuo X., Liao W., Ma C., He J., Chen Z. (2019). Characteristics of the sludge filterability and microbial composition in PAC hybrid MBR: Effect of PAC replenishment ratio. Biochem. Eng. J..

[44] Zhang M., Hong H., Lin H., Shen L., Yu H., Ma G., Chen J., Liao B.-Q. (2018). Mechanistic insights into alginate fouling caused by calcium ions based on terahertz time-domain spectra analyses and DFT calculations. Water Res..

[45] Salanitro J.P., Johnson P.C., Spinnler G.E., Maner P.M., Wisniewski H.L., Bruce C. (2000). Field-scale demonstration of enhanced MTBE bioremediation through aquifer bioaugmentation and oxygenation. Environ. Sci. Technol..

[46] Yang X., Song H., Lu J., Fu D., Cheng B. (2010). Influence of diatomite addition on membrane fouling and performance in a submerged membrane bioreactor. Bioresour. Technol..

[47] Wu M., Chen Y., Lin H., Zhao L., Shen L., Li R., Xu Y., Hong H., He Y. (2020). Membrane fouling caused by biological foams in a submerged membrane bioreactor: Mechanism insights. Water Res..

[48] Alizad Oghyanous F., Etemadi H., Yegani R. (2020). Foaming control and determination of biokinetic coefficients in membrane bioreactor system under various organic loading rate and sludge retention time. Biochem. Eng. J..

[49] Bezirgiannidis A., Marinakis N., Ntougias S., Melidis P. (2018). Membrane bioreactor performance during processing of a low carbon to nitrogen ratio municipal wastewater. Environ. Process.

[50] Palmarin M.J., Young S. (2019). The effects of biocarriers on the mixed liquor characteristics, extracellular polymeric substances, and fouling rates of a hybrid membrane bioreactor. Biochem. Eng. J..

[51] Gharibian S., Hazrati H. (2022). Towards practical integration of MBR with electrochemical AOP: Improved biodegradability of real pharmaceutical wastewater and fouling mitigation. Water Res..

[52] Kumar M., Adham S.S., Pearce W.R. (2006). Investigation of seawater reverse osmosis fouling and its relationship to pretreatment type. Environ. Sci. Technol..

[53] Kim I.S., Jang N. (2006). The effect of calcium on the membrane biofouling in the membrane bioreactor (MBR). Water Res..

[54] Croué J.P., Benedetti M.F., Violleau D., Leenheer J.A. (2003). Characterization and copper binding of humic and nonhumic organic matter isolated from the south platte river:  Evidence for the presence of nitrogenous binding site. Environ. Sci. Technol..

[55] Liu Z., Zhang C., Wang L., He J., Li B., Zhang Y., Xing X.H. (2015). Effects of furan derivatives on biohydrogen fermentation from wet steam-exploded cornstalk and its microbial community. Bioresour. Technol..

[56] Huang W., She Z., Gao M., Wang Q., Jin C., Zhao Y., Guo L. (2019). Effect of anaerobic/aerobic duration on nitrogen removal and microbial community in a simultaneous partial nitrification and denitrification system under low salinity. Sci. Total Environ..

[57] Han X., Wang Z., Ma J., Zhu C., Li Y., Wu Z. (2015). Membrane bioreactors fed with different COD/N ratio wastewater: Impacts on microbial community, microbial products, and membrane fouling. Environ. Sci. Pollut. Res..

[58] Liu Y., Liu Q., Li J., Ngo H.H., Guo W., Hu J., Gao M., Wang Q., Hou Y. (2018). Effect of magnetic powder on membrane fouling mitigation and microbial community/composition in membrane bioreactors (MBRs) for municipal wastewater treatment. Bioresour. Technol..

[59] Lu H., Chandran K., Stensel D. (2014). Microbial ecology of denitrification in biological wastewater treatment. Water Res..

[60] Ouyang E., Liu Y., Ouyang J., Wang X. (2019). Effects of different wastewater characteristics and treatment techniques on the bacterial community structure in three pharmaceutical wastewater treatment systems. Environ. Technol..

[61] Kozak M., Cırık K., Dolaz M., Başak S. (2021). Evaluation of textile wastewater treatment in sequential anaerobic moving bed bioreactor—aerobic membrane bioreactor. Process Biochem..

[62] Huang X., Xing Y.X., Wang H.J., Dai Z.Y., Chen T.T. (2022). Nitrogen advanced treatment of urban sewage by denitrification deep-bed filter: Removal performance and metabolic pathway. Front. Microbiol..

[63] Zhang Y., Wang K.T., Jiang W.L., He J.Y., Wang H., Li B., Gao M. (2020). Black odorous water concentrating by forward osmosis (FO) with aquaporin biomimetic membranes: Pollutants concentrating and membrane fouling characteristics. Chem. Eng. J..

[64] Zhang X.L., Li R.X., Song J., Ren Y., Luo X., Li Y., Li X., Li T., Wang X., Zhou Q. (2021). Combined phyto-microbial-electrochemical system enhanced the removal of petroleum hydrocarbons from soil: A profundity remediation strategy. J. Hazard. Mater.

[65] Jiang B., Zeng Q., Hou Y., Li H., Liu J., Xu J., Shi S., Ma F. (2020). Impacts of long-term electric field applied on the membrane fouling mitigation and shifts of microbial communities in EMBR for treating phenol wastewater. Sci. Total Environ..

