# Supplementary material for: Biochar Addition in Membrane Bioreactor Enables Membrane Fouling Alleviation and Nitrogen Removal Improvement for Low C/N Municipal Wastewater Treatment
Source: Membranes (Basel). 2023 Feb 4;13(2):194. doi: 10.3390/membranes13020194 (PMC9960794; doi:10.3390/membranes13020194)
Supplement: Supplementary file 1 [file membranes-13-00194-s001.zip › membranes-2182970-supplementary.pdf]

membranes

MDPI

Supplementary Materials

Biochar Addition in Membrane Bioreactor Enables Membrane Fouling Alleviation and Nitrogen Removal Improvement for Low C/N Municipal Wastewater Treatment

Kanming Wang [1,2], Qiaoqiao Ye [2], Yuxiang Shen [2], Yajing Wang [2], Qiankun Hong [2], Chenlong Zhang [3], Min Liu [1] and Hongyu Wang [2,*]

[1] College of Architecture and Environment, Sichuan University, Chengdu 610000, China
[2] College of Environment, Zhejiang University of Technology, Hangzhou 310014, China
[3] Ningbo Communications Planning Institute Co., LTD, Ningbo 315100, China
* Correspondence: hywang@zjut.edu.cn

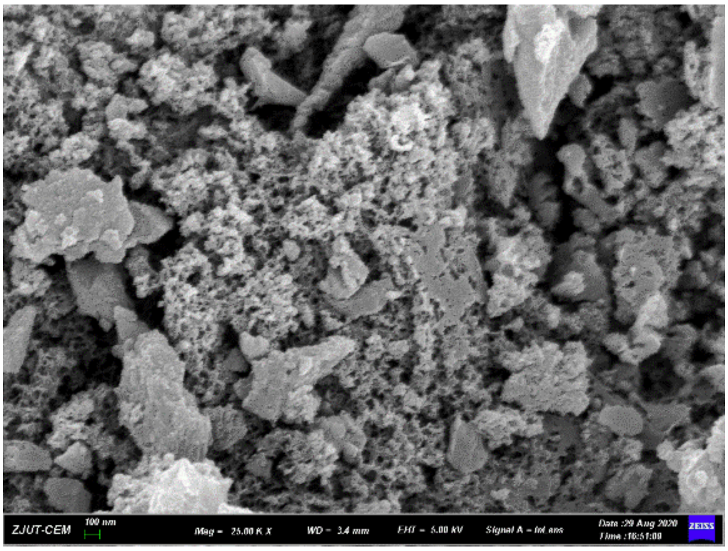

Figure S1. Scanning electron microscopic (SEM) images of biochar.

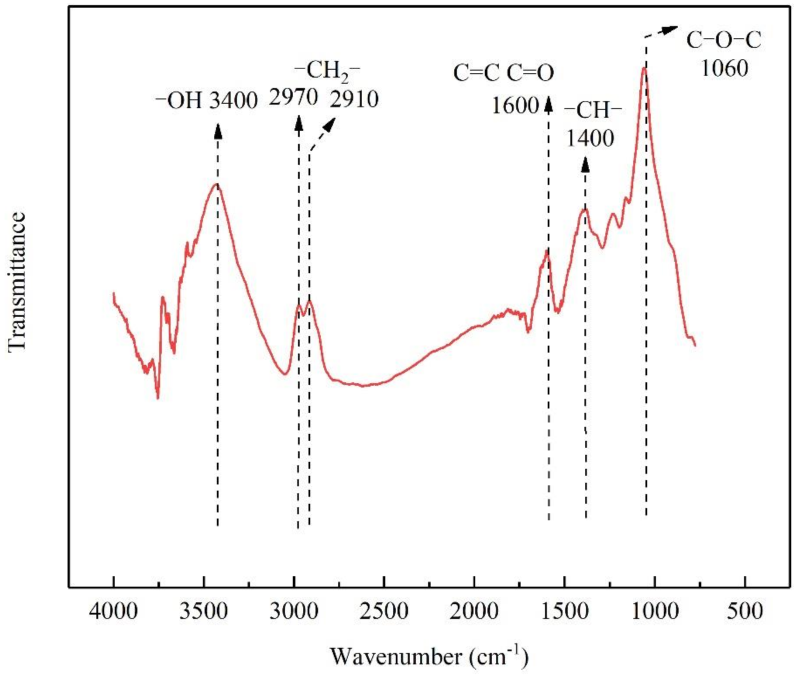

Figure S2. FTIR spectra and spectroscopic assignment of biochar.

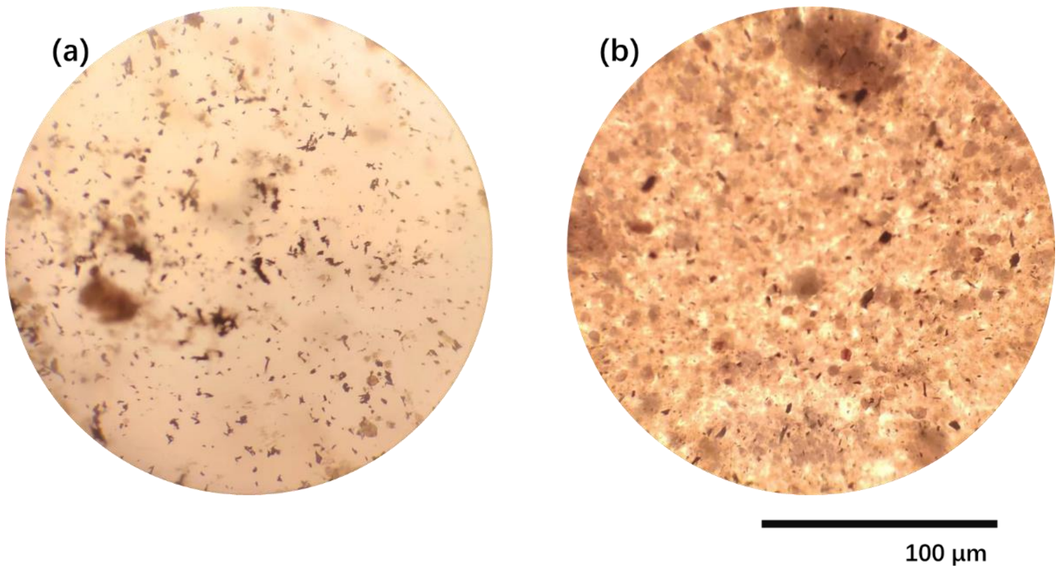

Figure S3. Microscope picture of (**a**) pristine BC and (**b**) the activated sludge with BC addition.

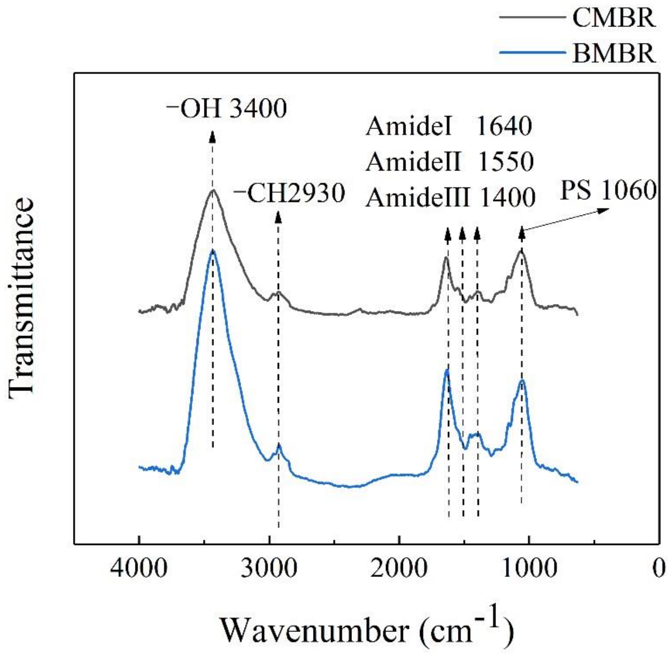

Figure S4. FTIR spectra and spectroscopic assignment of the polluted membrane up to 35 kPa of conventional MBR (CMBR) and biochar MBR (BMBR).

Table S1. Component of the synthetic wastewater.

**Macronutrient Solution**		**Micronutrient Solution**	
Component	Concentration (mg/L)	Component	Concentration (mg/L)
CH_3COONa	575.8	H_3BO_3	23.8
NH_4Cl	97.0	$CoCl_2·6H_2O$	23.8
Peptone	28.7	$CuSO_4·5H_2O$	7.1
KH_2PO_4	15.2	$FeCl_3·6H_2O$	238.1
Yeast extract	11.5	$MnCl_2·2H_2O$	26.2
$MgSO_4$	9.3	$NaMo_7O_{24}·2H_2O$	16.7
$MnSO_4$	1.1	$ZnSO_4·7H_2O$	23.8
$FeSO_4$	0.2	KI	7.1
		$NiCl_2$	14.3

Table S2. BET surface area, micropore area, pore volume and pore diameter of biochar.

	**BET Surface Area (m^2/g)**	**Micropore Area (m^2/g)**	**Pore Volume (cm^3/g)**	**Pore Diameter (μm)**
Biochar	645.667	322.584	0.243	3.814

Table S3. Treatment performance data and statistical results.

**COD**	Effluent (mg/L)					Average (mg/L)	*p* value	Removal (%)
Days	4	12	20	28	32			
CMBR	8.93	9.17	9.56	10.36	11.18	9.8	<0.05	96.4
BMBR	4.53	4.63	5.96	6.15	6.23	5.5		97.9
**NH_4^+-N**	Effluent (mg/L)					Average (mg/L)	*p* value	Removal (%)
Days	4	12	20	28	32			
CMBR	0.89	0.85	0.87	0.81	0.78	0.8	>0.05	98.22
BMBR	0.75	0.69	0.72	0.71	0.62	0.7		98.44
**NO_3^--N**	Effluent (mg/L)					Average (mg/L)	*p* value	Removal (%)
Days	4	12	20	28	32			
CMBR	18.95	20.30	21.59	21.89	22.77	21.1	<0.05	-
BMBR	14.95	16.24	16.61	17.75	17.95	16.7		-
**TN**	Effluent (mg/L)					Average (mg/L)	*p* value	Removal (%)
Days	4	12	20	28	32			
CMBR	19.84	21.15	22.46	22.71	23.54	21.9	<0.05	62.3
BMBR	15.70	16.93	17.34	18.46	18.57	17.4		70.1

Table S4. Amount of COD, DOC, SCFAs released from biochar.

**Parameter**		**Concentration (mg/g)**
COD		7.0 ± 0.5
DOC		6.8 ± 0.7
VFAs	Acetic acid	6.1 ± 0.6
	Propionic acid	5.0 ± 0.9

Table S5. Characterization of mixed liquor suspension data.

**SVI (mL/g)**								
Days	4	8	12	16	20	24	28	32
**CMBR**	88.87	93.39	96.53	101.18	105.69	108.38	116.12	128.10
**BMBR**	74.38	80.07	72.46	87.18	90.52	85.91	90.22	90.53
**SV_{30} (%)**								
Days	4	8	12	16	20	24	28	32
**CMBR**	76.0	84.0	86.0	76.0	86.0	96.0	98.0	98.0
**BMBR**	72.0	74.0	65.6	75.0	84.0	86.0	88.0	87.0
**Particle Size (μm)**								
Days	8	16	24	32				
**CMBR**	75.8	77.2	71.8	67.7				
**BMBR**	96.3	92.4	101.8	113.5				
**Zeta potential (–mV)**								
Days	8	16	24	32				
**CMBR**	17.5	18.7	19.4	20.6				
**BMBR**	17.2	18.5	18.9	19.4				
